# Depletion of neutrophils in a protective model of pulmonary cryptococcosis results in increased IL-17A production by gamma/delta T cells

**DOI:** 10.1186/1471-2172-13-65

**Published:** 2012-12-07

**Authors:** Karen L Wozniak, Jay K Kolls, Floyd L Wormley

**Affiliations:** 1 Department Biology, The University of Texas at San Antonio, San Antonio, TX 78249-0062, USA; 2 The South Texas Center for Emerging Infectious Diseases, The University of Texas at San Antonio, San Antonio, TX, USA; 3 Department of Pediatrics, Children’s Hospital of Pittsburgh of UPMC, Pittsburgh, PA, USA

**Keywords:** Gamma-delta T cells, *Cryptococcus neoformans*, IL-17A, Pulmonary

## Abstract

Protective responses in mice immunized with an interferon-gamma producing strain of *Cryptococcus neoformans*, H99γ, are associated with IL-17A production by neutrophils. Neutrophil depletion in H99γ-immunized mice did not affect pulmonary fungal burden, indicating that neutrophils are not required for clearance. However, we observed an increase in IL-17A in the lungs of neutrophil-depleted H99γ infected mice, which corresponded to an increase in IL-17A^+^ γδ^+^ T cells. Moreover, we observed increased IL-17A^+^/ CD3^+^ cells and IL-17A^+^/γδ^+^ cells, but decreased IL-17A^+^/Ly6G^+^ neutrophils in the lungs of IL-17 receptor (R)A deficient mice compared to wild-type mice. Increased production of IL-17A in neutropenic mice coincided with increased IL-6 and CXCL1, but not Th17 inducing cytokines TGF-β, IL-21 and IL-23. Concurrent depletion of neutrophils and γδ^+^ T cells reduced IL-17A levels. Our results suggest that γδ^+^ T cells mediate significant IL-17A production in neutropenic mice during the protective response to *C*. *neoformans* infection.

## Background

*Cryptococcus neoformans* is an opportunistic fungal pathogen that primarily affects patients with impaired T cell-mediated immunity. Clinically, symptomatic cryptococcal infections have been observed in individuals with AIDS, solid-organ transplant recipients receiving immunosuppressive medicines to prevent organ rejection, and patients taking chemotherapeutic drugs [1-5]. Experimental studies in mice have suggested that protection against cryptococcosis is primarily associated with the induction of Th1-type anti-cryptococcal cell-mediated immune responses [6-13]. In addition, Th17-type immune responses have been shown to be important for protection against multiple fungal pathogens [14-18]. However, the specific function of IL-17A and/or Th17-type immune responses in mediating protection against cryptococcal infection is uncertain [19-23].

Previously, we have shown that mice immunized with an interferon-gamma (IFN-γ) producing strain of *Cryptococcus neoformans*, denoted H99γ, were completely protected against subsequent challenge with wild-type cryptococci [11,12]. Protection was associated with the induction of Th1-type and pro-inflammatory cytokine responses and IL-17A production early during infection in protected animals [11,12]. While protection against *C*. *neoformans* strain H99γ required intact Th1-type cytokine responses, mice depleted of IL-17A and IL-17 receptor (R) A deficient (IL-17RA^−/−^) mice were able to survive acute infection with *C*. *neoformans* strain H99γ and no evidence of H99γ dissemination to the brain was observed [24]. Furthermore, IL-17RA^−/−^ mice immunized with *C*. *neoformans* strain H99γ were able to resolve a subsequent pulmonary challenge with wild-type *C*. *neoformans* strain H99. Nonetheless, some surviving IL-17RA^−/−^ mice exhibited evidence of dissemination of *C*. *neoformans* to the brain that was not observed in their immune competent counterparts, suggesting that prevention of dissemination is an important protective feature of IL-17A during cryptococcosis [24]. Our prior studies using intracellular cytokine staining followed by flow cytometric analysis suggested that the primary producers of IL-17A in our model system were neutrophils rather than Th17-type CD4^+^ T cells [24]. Furthermore, the IL-17A produced in our model of cryptococcal infection was not proceeded or accompanied by the production of cytokines that typically initiate Th17-type responses (i.e., TGF-β, IL-21, or IL-23) [12]. This is not unique, as other investigators have observed IL-17A production by neutrophils in other model systems [25-27]. Also, IL-17A production by multiple cell types including CD8^+^ T cells, γδ^+^ T cells, NK cells, and NKT cells have been demonstrated [25,28-37]. In the current studies, we further explored the role of neutrophils and IL-17A production in mice during infection with *C*. *neoformans* strain H99γ. Interestingly, depletion of neutrophils in mice infected with *C*. *neoformans* strain H99γ resulted in a significant increase of IL-17A in lung homogenates, which necessitated a search for alternate sources of IL-17A in neutropenic mice. The eventual depletion of neutrophils in combination with other cell types led to the identification of γδ^+^ T cells as a source of IL-17A production during pulmonary infection with *C*. *neoformans* strain H99γ.

## Results

### Depletion of neutrophils in mice infected with *C*. *neoformans* strain H99γ leads to increased IL-17A in lung homogenates

**Figure 1:**
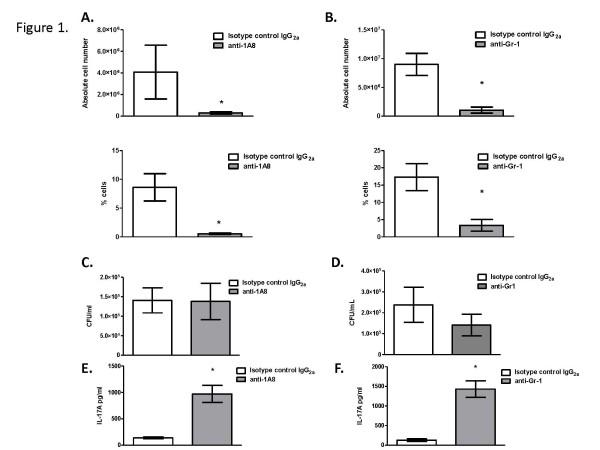
**Neutrophil depletion does not influence fungal burden but is associated with increased pulmonary levels of IL-17A during infection with *C. neoformans *strain H99γ.** BALB/c mice received an intranasal inoculum of 1 × 10^4^ CFU of *C. neoformans* strain H99γ in 50 μl of sterile PBS. Prior to and during infection, mice were treated with isotype control antibody (white bars) or with either anti-1A8 antibody or anti-Gr1 antibody (gray bars). Lungs were excised at day 7 post-inoculation, and neutrophil depletion, absolute numbers and percentages (**A**, **B**), pulmonary cryptococcal burden (**C**, **D**) and IL-17A production (**E**, **F**) quantified. Asterisks (*) indicate where significant differences (*P* < 0.05) were observed in neutrophil depleted mice compared to isotype-control treated mice following infection with *C. neoformans* strain H99γ. Fungal burden results are expressed as mean CFU per milliliter ± standard errors of the means. Data are cumulative of four experiments using 4 mice per group.

Our previous work employing intracellular cytokine staining followed by flow cytometric analysis suggested that neutrophils were the primary leukocyte source of IL-17A in mice infected with *C*. *neoformans* strain H99γ [24]. Therefore, we sought to determine the effect of neutrophil depletion on IL-17A production in the lungs of mice during infection with *C*. *neoformans* strain H99γ. Mice were depleted of neutrophils using two different neutrophil depletion antibodies, the anti-Gr1 antibody (clone RB6-8C5) and the anti-Ly6G antibody (clone 1A8), and control animals were treated with isotype control antibody beginning 24 hours prior to infection and every 48 hours thereafter. Total leukocytes were isolated from lung digests on day 7 post-infection to confirm neutrophil depletion and to phenotype the local leukocyte population. This time point was chosen because it is the time point at which pulmonary IL-17A production is at its peak during infection with *C*. *neoformans* strain H99γ [24]. Additionally, protein homogenates were prepared from lung tissues on day 7 post-infection to evaluate pulmonary IL-17A cytokine production and fungal burden in neutrophil depleted mice compared to isotype control antibody treated animals. Each depletion protocol implemented resulted in a successful depletion of both the absolute cell numbers and percentage of neutrophils present in the lungs compared to isotype control antibody treated mice (Figure 1A and B). Following neutrophil depletion with either antibody, fungal burden was not significantly different compared to that observed in isotype control antibody treated animals at day 7 post-inoculation (Figure 1C and D), as observed by previous investigators [38]. Interestingly, pulmonary homogenates of mice depleted of neutrophils by either antibody had significantly higher IL-17A present compared to mice treated with isotype control antibody (Figure 1E and F). While this result seemed counterintuitive, it is not unique and has been observed in other model systems during neutrophil depletion [26,39]. Previous studies have suggested that IL-10 production by neutrophils may lead to an inhibition of IL-17A production in the lungs [40]. However, we observed no significant difference in IL-10 present within lung homogenates derived from isotype control antibody treated mice in comparison to that observed in neutrophil depleted mice on day 7 post-inoculation (11.64 pg/ml ± 1.36 and 12.58 pg/ml ± 0.94, in isotype control antibody treated and clone 1A8 treated mice, respectively). Due to its cross-reactivity to the Ly6C antigen, the anti-Gr1 antibody depleted not only neutrophils but also CD8^+^ T cells (data not shown), as seen in studies by other investigators [41]. In contrast, the 1A8 clone was observed to specifically deplete neutrophils. Other leukocyte populations examined following 1A8 neutrophil depletion (CD4^+^ T cells, CD8^+^ T cells, macrophages, dendritic cells, regulatory T cells, natural killer T cells, γδ^+^ T cells, natural killer cells, eosinophils, and B cells) were not affected (data not shown). Therefore, we elected to perform all further experiments using the 1A8 antibody for neutrophil depletion.

### Determination of IL-17A-producing population in the absence of neutrophils

**Figure 2:**
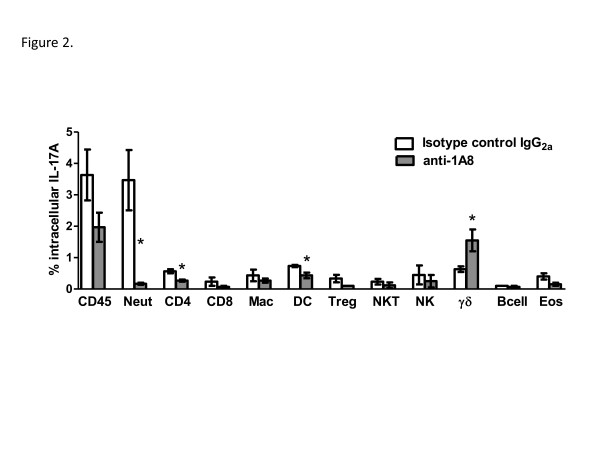
**Lung γδ**^**+**^**T cells are the predominant leukocyte population expressing IL-17A during pulmonary infection with *C. neoformans *strain H99γ during neutrophil depletion.** BALB/c mice received an intranasal inoculum of 1 × 10^4^ CFU of *C. neoformans* strain H99γ in 50 μl of sterile PBS. Mice were treated with isotype control antibody (white bars) or with anti-1A8 antibody (gray bars). The lungs were excised at day 7 post-inoculation and a single cell suspension generated using enzymatic digestion. The leukocytes were stained with anti-mouse antibodies (CD45, 1A8 (Neut), CD4, CD8, F4/80 (Mac), CD11b/CD11c (DC), CD4/Fox3p (Treg), CD3/NKp46 (NKT), NKp46/CD45 (NK), γδ/CD45 (γδ^+^ T cells), CD19 (B cell), SiglecF/CD11b (Eosinophil), fixed, permeabilized, and incubated with anti-IL-17A antibodies and evaluated by flow cytometry. Flow cytometry data are cumulative results of three independent experiments using pooled leukocytes from 4 mice per group per experiment. Results shown are the percentage of leukocytes expressing the indicated surface markers and IL-17A. Asterisks (*) indicate where significant differences (*P* < 0.05) were observed between isotype control-treated and anti-1A8-treated mice infected with *C. neoformans* strain H99γ.

We sought to determine the population responsible for the increased production of IL-17A in the lungs of neutrophil depleted mice on day 7 post-infection with *C*. *neoformans* strain H99γ. Total leukocytes were isolated from lung tissues, and the leukocyte subpopulations were characterized for intracellular IL-17A expression by flow cytometry. Leukocyte populations examined included neutrophils, CD4^+^ T cells, CD8^+^ T cells, macrophages, dendritic cells, regulatory T cells, natural killer T cells, natural killer cells, γδ^+^ T cells, B cells, and eosinophils. Figure 2 demonstrates that the only cell type with increased intracellular IL-17A expression during neutrophil depletion was the γδ^+^ T cell population. As expected, the percentage of IL-17A^+^ neutrophils was significantly reduced in neutrophil depleted mice, and other leukocyte subsets including CD4^+^ T cells and dendritic cells had significantly reduced intracellular staining for IL-17A following neutrophil depletion (Figure 2).

### Ex vivo IL-17 production in γδ^+^ T cells from neutrophil depleted mice

**Figure 3:**
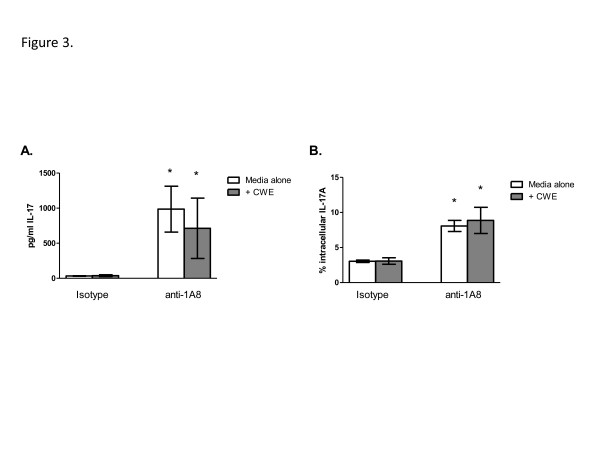
**Ex-vivo IL-17A production from γδ**^**+**^**T cells following neutrophil depletion.** BALB/c mice received an intranasal inoculum of 1x10^4^ CFU of *C. neoformans* strain H99γ in 50 μl of sterile PBS. Mice were treated with isotype control antibody or with anti-1A8 antibody. The lungs were excised at day 7 post-inoculation and a single cell suspension generated using enzymatic digestion. The leukocytes were isolated and then further purified for γδ^+^ T cells. The γδ^+^ T cells were plated at 1 × 10^5^ cells/well and incubated for 24 h at 37°C, 5% CO_2_ with media alone (white bars) or with *C. neoformans* cell wall extract (CWE) (gray bars). Cell supernatants were removed and examined for IL-17A by ELISA. Asterisks (*) indicate where significant differences (*P* < 0.05) were observed between γδ^+^ T cells from mice treated with isotype control antibody compared to those from mice treated with the 1A8 neutrophil depletion antibody. Data are cumulative of three separate experiments.

In order to confirm our observation that γδ^+^ T cells had increased IL-17A production in mice depleted of neutrophils, we isolated γδ^+^ T cells from H99γ-infected mice treated with either isotype control antibody or the 1A8 neutrophil-depleting antibody on day 7 post-infection. Once isolated, γδ^+^ T cells were plated in tissue culture wells with and without cryptococcal antigen for 24 h. Following incubation, supernatants were harvested and examined for IL-17A by ELISA, and cells were examined for intracellular IL-17A by flow cytometry. Results showed that IL-17A protein levels (Figure 3A) as well as intracellular IL-17A (Figure 3B) were significantly increased in γδ^+^ T cells from neutrophil-depleted mice compared to isotype control-treated mice, therefore confirming our previous observations (Figure 2).

### Cytokine production and intracellular IL-17A expression in mice deficient in IL-17RA signaling

**Figure 4:**
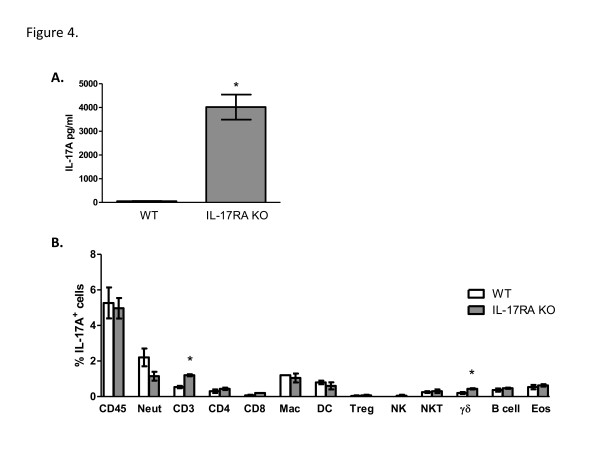
**Induction of IL-17A production during *C. neoformans *strain H99γ infection requires signaling through the IL-17A receptor.** BALB/c (white bars) and IL-17RA KO (gray bars) mice were given an intranasal inoculation with *C. neoformans* strain H99γ. Lungs were excised at day 7 post-inoculation, and pulmonary cytokine production quantified (**A**) and intracellular IL-17A production was determined in leukocyte populations (**B**). The lungs were excised at day 7 post-inoculation and a single cell suspension generated using enzymatic digestion. The leukocytes were stained with anti-mouse antibodies (CD45, 1A8 (Neut), CD4, CD8, F4/80 (Mac), CD11b/CD11c (DC), CD4/Fox3p (Treg), CD3/NKp46 (NKT), NKp46/CD45 (NK), γδ/CD45 (γδ^+^ T cells), CD19 (B cell), SiglecF/CD11b (Eosinophil), fixed, permeabilized, and incubated with anti-mouse antibodies specific for IL-17A and quantified by flow cytometry. Asterisks (*) indicate where significant differences (*P* < 0.05) were observed between WT and IL-17RA KO mice infected with *C. neoformans* strain H99γ. Data are cumulative of three experiments using 3 mice per group.

We hypothesized that intracellular staining for IL-17A may be indicative of which cells produce IL-17A; therefore, we wanted to rule out the possibility that our intracellular staining may be detecting the recipients of IL-17A. Consequently, we evaluated IL-17A production in IL-17RA^−/−^ mice compared to wild-type mice on day 7 following infection with *C*. *neoformans* strain H99γ. We rationalized that increased cellular production of IL-17A will be observed but IL-17A uptake will not be detected in IL-17RA^−/−^ mice, thus providing some insight into which cells are the producers and/or the target for IL-17A in our model system. We observed a significant increase in IL-17A within lung homogenates derived from IL-17RA^−/−^ mice compared to wild-type mice on day 7 post-infection with *C*. *neoformans* strain H99γ (Figure 4A). Total leukocytes were also isolated from lung tissues, and the leukocyte subpopulations characterized for intracellular IL-17A expression by flow cytometry. We observed an overall decrease, though not reaching statistical significance, in the percentage of IL-17A^+^ neutrophils in IL-17RA^−/−^ mice compared to wild-type mice. However, significantly increased IL-17A^+^/CD3^+^ and IL-17A^+^/γδ^+^ T cell populations were also observed in the lungs of IL-17RA^−/−^ mice compared to wild-type mice on day 7 post-infection (Figure 4B). No other significant differences were observed.

### Cytokine and chemokine production in mice depleted of neutrophils

**Figure 5:**
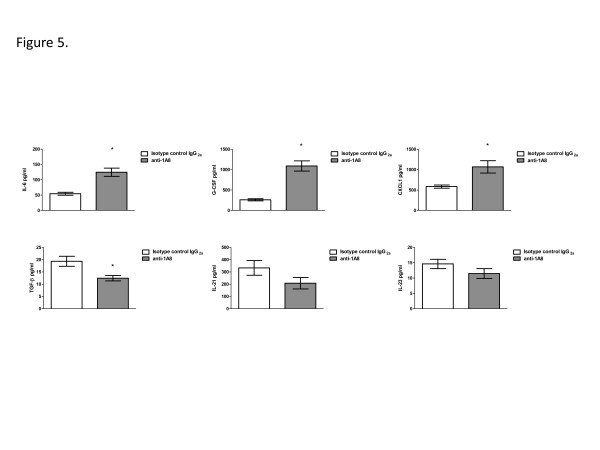
**Neutrophil depletion during pulmonary infection with *C. neoformans *strain H99γ results in significant production of neutrophil chemoattractant cytokines, but little change in Th17-type cytokine production in the lungs.** BALB/c mice were given an intranasal inoculation with *C. neoformans* strain H99γ. Mice were treated with isotype control antibody (white bars) or with anti-1A8 antibody (gray bars). Lung homogenates were prepared from lungs excised on day 7 post-inoculation and assayed for IL-6, G-CSF, CXCL1, TGF-β, IL-21, and IL-23 cytokine production. Data are cumulative of three experiments using 5 mice each per group. Asterisks (*) indicate where significant differences (*P* < 0.05) were observed.

We posited that other cytokines/chemokines known to induce neutrophil chemotaxis may be increased in the lungs of neutrophil depleted mice following infection with H99γ, since recruitment of neutrophils occurs during infection with H99γ. Therefore, we determined the expression of cytokines involved in neutrophil recruitment, including IL-6, G-CSF, and CXCL1, within the lungs of neutrophil depleted and isotype control antibody treated mice on day 7 post-infection with *C*. *neoformans* strain H99γ. As shown in Figure 5A, IL-6, G-CSF, and CXCL1 were significantly increased in the lung homogenates of neutrophil depleted mice compared to lung homogenates of isotype control antibody treated mice, suggesting an attempt by the host to recruit neutrophils to the site of infection. Cytokines typically associated with a Th17-type response, IL-21, IL-23, and TGF-β, were also investigated to support our previous observation that the IL-17A response to this infection is part of an overall chemokine response and not associated with a classical Th17-type response. IL-21 and IL-23 levels were not significantly changed during neutrophil depletion, and TGF-β was significantly decreased in neutrophil depleted mice in comparison to isotype control antibody treated mice (Figure 5B).

### Depletion of additional leukocyte populations during neutrophil depletion

**Figure 6:**
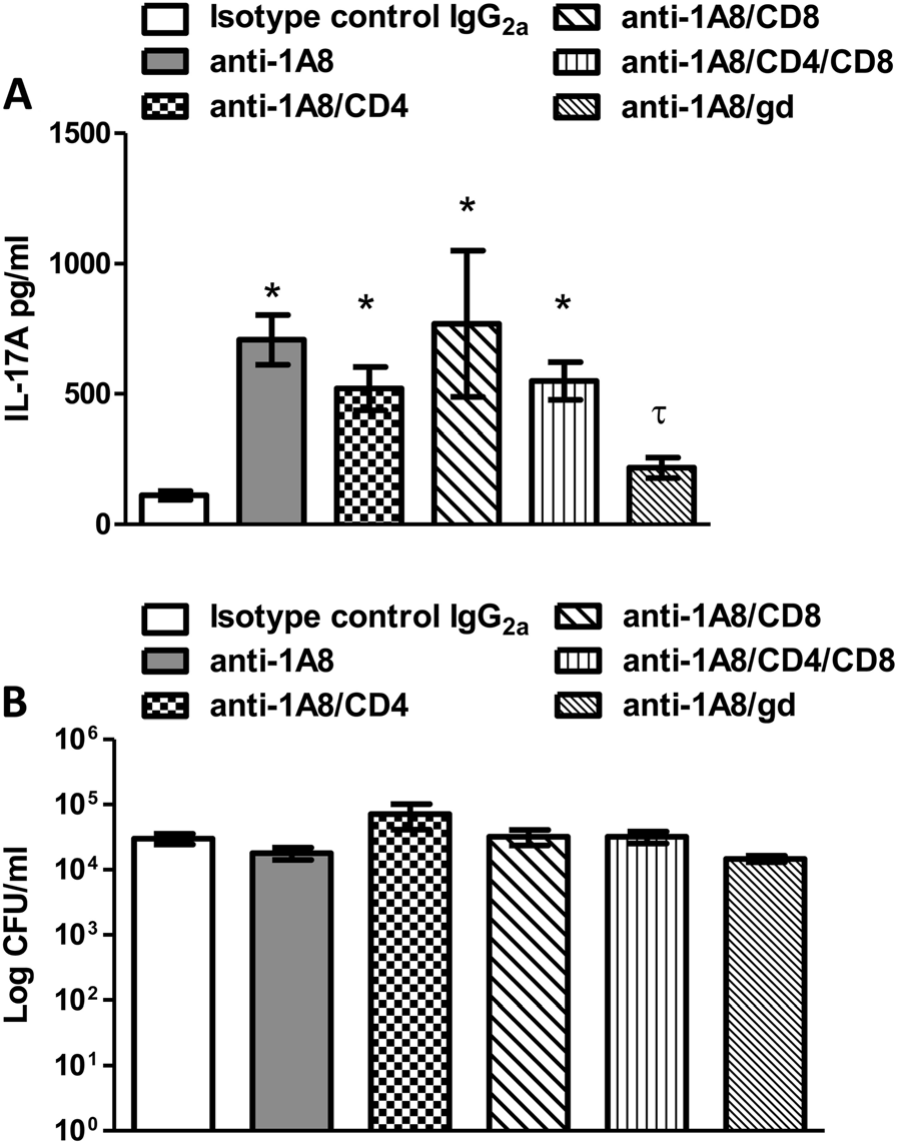
**Neutrophil and γδ**^**+**^**T cell depletion does not affect fungal burden, but does abrogate IL-17A production in mice infected with *C. neoformans *strain H99γ.** BALB/c mice received an intranasal inoculum of 1 × 10^4^ CFU of *C. neoformans* strain H99γ in 50 μl of sterile PBS. Prior to and during infection, mice were treated with isotype control antibody (white bars) or with anti-1A8 antibody (gray bars), anti-1A8/CD4 (checkered bars), anti-1A8/CD8 (slashed bars), anti-1A8/CD4/CD8 (striped bars), and anti-1A8/γδ (thin slashed bars). Lungs were excised at day 7 post-inoculation, and IL-17A (**A**) and pulmonary cryptococcal burden (**B**) quantified. Asterisks (*) indicate where significant increases in depleted mice compared to isotype-control treated mice (*P* < 0.05), tau (τ) indicates significant decreases in depleted mice compared to neutrophil (1A8) depleted mice (*P* < 0.05) following infection with *C. neoformans* strain H99γ. Fungal burden results are expressed as mean CFU per milliliter ± standard errors of the mean (SEM), and IL-17A results are expressed as the mean pg/ml ± SEM. Data are cumulative of three experiments using 4 mice per group.

Our observation that CD3^+^ and γδ^+^ cells have significantly increased intracellular IL-17A production during neutrophil depletion led us to examine these populations more closely. For this, mice were depleted of neutrophils alone, or neutrophils in combination with CD4^+^ T cells and/or CD8^+^ T cells, and γδ^+^ T cells and given an experimental pulmonary infection with *C*. *neoformans* strain H99γ. IL-17A production and pulmonary fungal burden was subsequently analyzed on day 7 post-inoculation. Depletion of neutrophils in combination with CD4^+^ and/or CD8^+^ T cell subsets did not affect IL-17A production in the lungs of infected mice compared to infected mice treated with isotype control antibody (Figure 6A). In contrast, depletion of neutrophils in combination with γδ^+^ T cells resulted in a significant reduction in pulmonary IL-17A compared to IL-17A detected in lung homogenates of mice depleted of neutrophils alone on day 7 post-infection (Figure 5A). Nonetheless, we observed no significant difference in pulmonary fungal burden in all depletion groups at day 7 post-infection (Figure 6B). Therefore, the γδ^+^ T cells appear to be responsible for the increased IL-17A detected in lung homogenates of neutropenic mice infected with *C*. *neoformans* strain H99γ.

## Discussion

Our results discussed herein show that γδ^+^ T cells produce significant quantities of IL-17A in mice depleted of neutrophils during pulmonary infection with *C*. *neoformans* strain H99γ. The production of IL-17A did not appear commensurate with the percentage of IL-17A^+^ γδ^+^ T cells observed in pulmonary tissues of *C*. *neoformans* strain H99γ infected mice. Nonetheless, dual depletion of neutrophils and γδ^+^ T cells resulted in a significant reduction in pulmonary IL-17A production compared to mice depleted of neutrophils alone. This may suggest that neutrophils negatively regulate the production of IL-17A from γδ^+^ T cells. Previous studies have suggested that IL-10 production by neutrophils may lead to an inhibition of IL-17A production in the lungs [40], however, we observed no evidence of this occurring in our model system. Alternatively, the presence of cytokines or other stimulants that promote IL-17A production by γδ^+^ T cells may be enhanced in the lungs of neutrophil depleted mice in our model system. Thus, neutrophils would only indirectly influence IL-17A production by γδ^+^ T cells. While we are uncertain of the mechanism, we are not the first to demonstrate a contribution by γδ^+^ T cells to IL-17A production [27,29-31,34,35], and these data provide us with additional insight into perhaps an underappreciated role of γδ^+^ T cells in cytokine production at the pulmonary mucosa. In our model of protection against pulmonary cryptococcosis using *C*. *neoformans* strain H99γ, IL-17A production appears to work in concert with Th1-type immune responses to mediate protection and prevent dissemination to the brain [11,12]. Previous experiments employing intracellular staining for IL-17A followed by flow cytometric analysis indicated neutrophils as the predominant source of IL-17A in the lung during protective anti-cryptococcal immune responses [24]. Neutrophils do not appear to be critical for clearance of pulmonary *C*. *neoformans* infections; indeed, depletion of neutrophils alone in mice was shown to prolong survival and lower fungal burden [38]. However, neutrophils are phagocytic, anti-fungal, and also participate in the production of cytokines that enhance protective anti-cryptococcal immune responses [41-44]. Quite possibly, other cell types assume a greater effector role against *C*. *neoformans* to compensate for the absence of neutrophils in the lung.

The γδ^+^ T cell is an often overlooked cell type in immunity, but recent reports indicate that these cells provide critical protection in response to a variety of infections [39,45]. Their role in protection and IL-17A production has also recently been shown in several different models [28,35,36], thus supporting these current findings. While these cells were originally thought to be a primordial innate T cell with no memory capabilities (reviewed in [46]), more recent studies have shown that γδ^+^ T cells actually can respond to pathogen challenge and may function as a bridge between innate and adaptive immune responses [45]. Recent studies with the fungal organism *Candida albicans* have shown that IL-17A production by γδ^+^ T cells is important in early protection [39]. However, a previous study examining γδ KO mice as well as depletion of γδ^+^ T cells during cryptococcal infection showed enhanced fungal clearance [39]. In our model, which uses a different strain of mice and *Cryptococcus*, however, depletion of γδ^+^ T cells did not affect pulmonary fungal burden at early time points. Future studies in our laboratory will address the role of γδ^+^ T cells in mediating long-term protection during acute infection with our protective H99γ strain and subsequent survival following challenge with wild-type *C*. *neoformans* strain H99.

We previously showed that immunization with *C*. *neoformans* strain H99γ induced protection against a subsequent intranasal challenge with wild-type *C*. *neoformans* strain H99 in mice depleted of CD4^+^ T cells and/or CD8^+^ T cells [47]. Clinically, we know that *C*. *neoformans* infection results in significant morbidity and mortality in immune compromised individuals with low CD4^+^ T cells [1-5]. Traditional “innate” cell populations, such as NK cells, NKT cells, LTi cells, and γδ^+^ T cells can mediate protective immunity in the presence or absence of traditional adaptive mechanisms [48-52]. Demonstrating that cell populations such as γδ^+^ T cells participate in protective anti-cryptococcal immune responses and uncovering a means to enhance these responses will be critical to the development of effective immune therapies and/or vaccines targeted for use in immunocompromised patient populations.

In these data presented herein, we used intracellular cytokine staining followed by flow cytometry analysis to determine the IL-17A-producing population of cells within mice given an experimental pulmonary infection with *C*. *neoformans* strain H99γ. However, we acknowledge that this technique may not adequately represent the entire picture since intracellular staining only allows us to identify the percentage of positively-stained cells, and therefore it is not quantitative. Therefore, it is possible that we may be underestimating the contributions of specific leukocyte populations as producers of IL-17A, since we cannot determine the specific amount of IL-17A produced by each individual cell type using intracellular staining alone. Also, intracellular staining may identify cells that are the target cells for the cytokine, in addition to cells producing the cytokine. Further, our ex vivo data using purified γδ^+^ T cells from H99γ-infected mice depleted of neutrophils verified their IL-17A production. Our results using mice deficient in IL-17RA signaling show that the percentage of IL-17A^+^ neutrophils were decreased by approximately 47% compared to wild-type mice during infection with *C*. *neoformans* strain H99γ, which supports this conclusion. In addition to γδ^+^ T cells, other CD3^+^ cells can also produce IL-17A, including invariant natural killer cells (iNK) and lymphoid tissue inducer cells (LTi) [53-55]. However, we examined these cell types and did not observe the presence of these cells in the lung during infection with *C*. *neoformans* strain H99γ (Wozniak & Wormley, unpublished observations).

## Conclusions

In conclusion, the data presented herein describe the identification of the γδ^+^ T cell as a significant source of IL-17A in the lungs of neutropenic mice during infection with *C*. *neoformans* strain H99γ. Depletion of these cells leads to abrogation of the enhanced IL-17A production observed upon depletion of neutrophils during infection with the protective *C*. *neoformans* strain H99γ. These γδ^+^ T cells may play an important role in leukocyte chemotaxis and the induction of protective immune responses to *C*. *neoformans*, especially in the absence of traditional adaptive immune responses. If this proves to be the case, these findings may have implications in the design of treatments or vaccines to combat cryptococcal disease. Ongoing studies are examining the role of these cells during challenge with wild-type *C*. *neoformans* strain H99, and their potential role in animals lacking all traditional adaptive immune cell types.

## Methods

### Mice

Female BALB/c (H-2^d^) (National Cancer Institute/Charles River Laboratories, Boston, MA and The Jackson Laboratory, Bar Harbor, ME) and IL-17 receptor A knock out (IL-17RA^−/−^) mice (a kind gift of Amgen, Inc. Thousand Oaks, CA), all on the BALB/c background with an average weight of 20–25 grams, were used throughout these studies. This study was carried out in strict accordance with the recommendations in the Guide for the Care and Use of Laboratory Animals of the National Institutes of Health. Mice were housed at The University of Texas at San Antonio Small Animal Laboratory Vivarium. These animal experiments were approved by The University of Texas at San Antonio Institutional Animal Care and Use Committee (IACUC), and mice were handled according to IACUC guidelines. All efforts were made to minimize animal suffering.

### Strains and media

*C*. *neoformans* strain H99γ (serotype A, Mat α, an interferon-gamma producing strain derived from *C*. *neoformans* H99 [11]) was recovered from 15% glycerol stocks stored at −80°C prior to use in the experiments described herein. The strain was maintained on yeast-extract-peptone-dextrose (YPD) media (1% yeast extract, 2% peptone, 2% dextrose, and 2% Bacto agar) supplemented with nourseothricin. Yeast cells were grown for 18–20 h at 30°C with shaking in YPD broth (Becton Dickinson and Company, Sparks, MD), collected by centrifugation, washed three times with sterile phosphate-buffered saline (PBS), and viable yeast quantified using trypan blue dye exclusion in a hemacytometer. For tissue culture, complete medium consisted of RPMI 1640 supplemented with 10% heat-inactivated fetal bovine serum, 2 mM L-glutamine, 100 U penicillin/ml, 100 μg of streptomycin/ml, and 50 mM 2-mercaptoethanol.

### Pulmonary infections

Pulmonary *C*. *neoformans* infections were initiated by nasal inhalation as previously described [12]. BALB/c mice were anesthetized with 2% isoflurane using a rodent anesthesia device (Eagle Eye Anesthesia, Jacksonville, FL) and then given a yeast inoculum of 1 × 10^4^ colony forming units (CFU) of *C*. *neoformans* strain H99γ in 50 μl of sterile PBS pipetted directly into the nares. The inocula used were verified by quantitative culture on YPD agar. The mice were fed ad libitum and were monitored by inspection twice daily. Mice were euthanized at specific time points post-inoculation by CO_2_ inhalation followed by cervical dislocation, and lung tissues were excised using aseptic technique. Tissues were homogenized in 1 ml of sterile PBS, followed by culture of 10-fold dilutions of each tissue on YPD agar supplemented with chloramphenicol (Mediatech, Inc., Herndon, VA). CFU were enumerated following incubation at 30°C for 48 h.

### Pulmonary leukocyte isolation

Lungs were excised at specific time points post-inoculation and digested enzymatically at 37°C for 30 minutes in 10 ml of digestion buffer (RPMI 1640 and 1 mg/ml of collagenase type IV [Sigma-Aldrich, St. Louis, MO.]) with intermittent (every 10 min) stomacher homogenizations. The enzymatically-digested tissues were then successively filtered through sterile nylon filters of various pore sizes (70 and 40 μm) (BD Biosciences) and washed with sterile HBSS to enrich for leukocytes. Erythrocytes were lysed by incubation in NH4Cl buffer (0.859% NH_4_Cl, 0.1% KHCO_3_, 0.0372% Na_2_EDTA [pH 7.4]; Sigma-Aldrich) for 3 minutes on ice followed by the addition of a 10-fold excess of PBS. The resulting leukocyte population was then collected by centrifugation (800 × *g*) for 5 minutes, washed twice with sterile PBS, resuspended in sterile PBS + 2% heat-inactivated fetal bovine serum (FACS buffer) and enumerated in a hemacytometer using trypan blue dye exclusion. Flow cytometric analysis was used to determine the percentage of each leukocyte population as well as the absolute number of total leukocytes (CD45^+^) within the lung cell suspension for standardization of hemacytometer counts.

### Cell depletions

For neutrophil depletion experiments, mice received either 200 μg anti-Gr1 antibody (clone RB6-8C5) (BioXCell) or 200 μg anti-Ly6G antibody (clone 1A8) (BioXCell) in a volume of 100 μl injected intraperitoneally beginning 24 hours post-inoculation and continuing every other day throughout the study. For depletion of gamma-delta T cells, mice received 100 μg anti-gamma-delta T cell receptor antibody (eBioscience) via the intraperitoneal route beginning two days prior to inoculation and continued weekly throughout the experiment. These concentrations and schedules were chosen following studies testing different dosages and schedules in our laboratory to determine the optimum dosage and schedule for each antibody (data not shown). Controls for neutrophil and γδ^+^ T cell depletions included mice treated with IgG_2a_ isotype control antibody (eBioscience Inc., San Diego, CA) via the intraperitoneal route. Mice were depleted of CD4^+^ and/or CD8^+^ T cell subsets via intraperitoneal administration of anti-CD4 (GK1.5, rat IgG_2b_) and anti-CD8α (2.43, rat IgG_2b_) antibodies (each from National Cell Culture Center, Minneapolis, MN). Each mouse received 200 μg of GK1.5 and/or 2.43 or control rat IgG_2b_ (eBioscience Inc.) antibodies in a volume of 100 μl PBS 48 h prior to infection and weekly thereafter. Cellular depletions were confirmed for each experiment by flow cytometry using antibodies that adhere to epitopes distinct from those adhered to by the depletion antibodies. Antibodies depleted approximately 95% of neutrophils, 98% of γδ^+^ T cells, 98% of CD4^+^ T cells, and 98% of CD8^+^ T cells (data not shown).

### Antibodies

For flow cytometry experiments, cells were incubated with CD16/CD32 (Fc Block™) (BD Biosciences, San Diego, CA) and the following antibodies conjugated to phycoerythrin (PE), allophycocyanin (APC), Alexa 647, or PECy7 were added: a cocktail of CD3, CD4, and CD8α; CD45, CD19, Siglec-F (BD Biosciences), 1A8, CD11c, CD11b, F4/80, NKp46, Fox3P, γδ, IL-17A, FcεR1α, CD117, CD34 (eBioscience Inc.), and F4/80 (Caltag Laoratories, Burlingame, CA).

### Flow cytometry

Standard methodology was employed for the direct immunofluorescence of pulmonary leukocytes. Briefly, in 96-well U-bottom plates, 100 μl containing 1 × 10^6^ cells in PBS + 2% FBS (FACS buffer) were incubated with 50 μl of Fc Block™ (BD Biosciences) diluted in FACS buffer for 5 minutes to block non-specific binding of antibodies to cellular Fc receptors. Subsequently, an optimal concentration of fluorochrome-conjugated antibodies (between 0.06-0.5 μg/1 × 10^6^ cells in 50 μl of FACS buffer) were added in various combinations to allow for dual or triple staining experiments, and plates were incubated for 30 minutes at 4°C. Following incubation, the cells were washed three times with FACS buffer and cells were fixed in 200 μl of 2% ultrapure formaldehyde (Polysciences, Inc., Warrington, PA) diluted in FACS buffer (fixation buffer). For intracellular staining, cells remained in fixation buffer for 10 min at room temperature. After fixation, the cells were washed and permeabilized with 0.1% saponin for 10 min at room temperature. While permeabilized, the cells were intracellularly stained with anti-IL-17A (eBioscience Inc.) and/or anti-Fox3P (regulatory T cell) (eBioscience Inc.) for 30 min at 4°C. Cells were then washed 3 times with 0.1% saponin and then resuspended in fixation buffer before flow cytometry was performed. Cells incubated with either FACS buffer alone or single fluorochrome-conjugated antibodies were used to determine positive staining and spillover/compensation calculations and the flow cytometer determined background fluorescence. The samples were analyzed using BD FACSArray software™ on a BD FACSArray flow cytometer (BD Biosciences). Dead cells were excluded on the basis of forward angle and 90° light scatter. For data analyses, 30,000 events (cells) were evaluated from a predominantly leukocytic population identified by backgating from CD45^+^-stained cells. The absolute number of total leukocytes was quantified by multiplying the total number of cells observed by hemacytometer counting by the percentage of CD45^+^ cells determined by flow cytometry. The absolute number of each leukocyte subset (1A8, F4/80^+^, CD11c^+^/CD11b^int^, CD19^+^, CD4^+^/CD3^+^, CD8^+^/CD3^+^, CD4^+^/Fox3p^+^, CD3^-^/NKp46^+^, CD3^+^/NKp46^+^, γδ^+^, Siglec-F^+^/CD11b^int^ was determined by multiplying the percentage of each gated population by the total number of CD45^+^ cells.

### γδ^+^ T cell ex vivo IL-17 production

Following leukocyte enrichment (see above), lung leukocytes from mice treated with either isotype control antibody or with anti-1A8 antibody were enriched for γδ^+^ T cells by positive selection using magnetic beads labeled with γδ antibody according to the manufacturer’s recommendations (Miltenyi Biotec, Auburn, CA). Cells were counted, and 1 × 10^5^ cells/well were plated in triplicate in 96-well plates, with either complete media alone or with 100 μg/ml *C*. *neoformans* cell wall extract (CWE) [13]. CWE was tested for endotoxin contamination before use, and levels were confirmed to be <1 EU/μg protein (data not shown). Cells were incubated at 37°C, 5% CO_2_ for 24 hr. Following incubation, cells were centrifuged and supernatants were removed for quantification of IL-17A by ELISA. Remaining cells were harvested and used for intracellular IL-17A staining by flow cytometry.

### Cytokine analysis

Cytokine levels in lung tissues were analyzed using the Bio-Plex Protein Array System (Luminex-based technology) (Bio-Rad Laboratories, Hercules, CA). Briefly, lung tissue was excised and homogenized in ice-cold sterile PBS (1 ml). An aliquot (50 μl) was taken to quantify the pulmonary fungal burden and an anti-protease buffer solution (1 ml) containing PBS, protease inhibitors (inhibiting cysteine, serine, and other metalloproteinases) and 0.05% Triton X-100 was added to the homogenate. Samples were then clarified by centrifugation (800 ×  g) for 5 minutes. Supernatants from pulmonary homogenates were assayed using the Bio-Plex Protein Array System (Bio-Rad Laboratories) for the presence of IL-6, IL-10, IL-17A, and granulocyte-colony stimulating factor (G-CSF) expression, as well as the chemokine keratinocyte-derived chemokine (KC) (CXCL1). ELISA assays were performed according to manufacturer’s instructions to measure TGF-β, IL-23 (R&D Systems), and IL-21 (BD Biosciences) on pulmonary homogenates, and IL-17A ELISA (R&D Systems) was used to measure IL-17A in cell culture supernatants.

### Statistical analysis

The unpaired Student’s *t* test (two-tailed) was used to analyze fungal burden, pulmonary cell populations, and cytokine/chemokine data using GraphPad Prism version 5.00 for Windows (GraphPad Prism Software, San Diego California USA). For multiple comparisons, a one-way ANOVA with the Tukey’s multiple comparison test was performed. Significant differences were defined as *P* < 0.05.

## Abbreviations

YPD: Yeast-extract-peptone-dextrose media.

## Competing interests

The authors declare that they have no competing interests.

## Authors’ contributions

KW and FW conceived and designed the experiments, KW carried out the experiments, JK contributed knock-out mice, KW, JK, and FW analyzed the data, and KW and FW wrote the manuscript. All authors read and approved the final manuscript.
